# Supplementary material for: Genetic Influence on the Peripheral Differentiation Signature of Vδ2+ γδ and CD4+ αβ T Cells in Adults
Source: Cells. 2021 Feb 11;10(2):373. doi: 10.3390/cells10020373 (PMC7918850; doi:10.3390/cells10020373)
Supplement: Supplementary file 1 [file cells-10-00373-s001.pdf]

Supplementary Table 1: Monoclonal antibody panel for flow cytometry

Specificity	Fluorophore	Clone
CD3	Alexa Fluor 700	UCHT-1
CD4	PE-Cy7	OKT4
CD8	APC-H7	SK1
CD16	BV711	3G8
CD27	APC	0323
CD28	PE	CD28.2
CD45RA	Pacific Blue	H100
γδ-TCR	Purified mouse Ab	11F2
Anti-mouse F(ab′)$_2$-Fragment	Pacific Orange	-
Vδ1-TCR	FITC	TS8.2
Vδ2-TCR	PerCP	B6
Dead cells	EMA	-

Supplementary Table 2: Configuration of the BD LSR II with customized filter settings

Laser wavelength	Laser power	Laser type	Optical filters		Spectral range [nm]	Fluorochrome
			Long pass [nm]	Band pass [nm]		
405 nm	25 mW	Vio Flame	685	700/13	693.5-706.5	BV711
			635	660/20	650-670	BV650
			600	610/20	600-620	BV605
			545	561/14	554-568	Pacific Orange
			-	450/50	425-475	Pacific Blue
488 nm	20 mW	Coherent Sapphire Solid State	735	780/60	750-810	PE-Cy7
			685	695/40	685-715	PerCP-Cy5.5
			655	670/14	663-677	PerCP
			600	610/20	600-620	EMA
			550	575/25	562.5-587.5	PE
			505	530/30	515-545	FITC
			-	488/10		SSC
633 nm	17 mW	JDS Uniphase HeNe	635	780/60	750-810	APC-H7
			710	730/45	710-752.2	Alexa Fluor 700
			-	660/20	650-670	APC

Supplementary Table 3: Cohort characteristics of twin pairs

Variable	Category	Samples	
		n	%
Gender (DZ)	Male	12	35.3
	Female	22	64.7
Gender (MZ)	Male	16	32.0
	Female	34	68.0
CMV serostatus (DZ)	Positive	27	79.4
	Negative	7	20.6
CMV serostatus (MZ)	Positive	34	68.0
	Negative	16	32.5
Age (DZ)	Median	50 years	
	Age range	43-77 years	
Age (MZ)	Median	46 years	
	Age range	41-64 years	

Supplementary Table 4: Spearman correlation for all differentiation phenotypes of CD4+, CD8+, Vδ1+ and Vδ2+ T cells for MZ and DZ twins.

Subset	Twins	Spearman r	p-value	95% CI	
CD4+CD27+CD28+CD45RA+	MZ	0,679	0,000	0,377	0,850
CD4+CD27+CD28+CD45RA-	MZ	0,870	< 0,0001	0,718	0,943
CD4+CD27+CD28-	MZ	0,701	< 0,0001	0,413	0,862
CD4+CD27-CD28+	MZ	0,646	0,001	0,326	0,833
CD4+CD27-CD28-CD45RA-	MZ	0,055	0,819	-0,409	0,496
CD4+CD27-CD28-CD45RA+	MZ	-0,050	0,835	-0,492	0,414
CD8+CD27+CD28+CD45RA+	MZ	0,594	0,002	0,248	0,805
CD8+CD27+CD28+CD45RA-	MZ	0,465	0,019	0,074	0,733
CD8+CD27+CD28-	MZ	0,382	0,059	-0,027	0,682
CD8+CD27-CD28+	MZ	0,775	< 0,0001	0,538	0,898
CD8+CD27-CD28-CD45RA-	MZ	0,679	0,000	0,378	0,851
CD8+CD27-CD28-CD45RA+	MZ	0,449	0,025	0,053	0,723
Vδ1+CD27+CD28+CD45RA+	MZ	0,286	0,197	-0,167	0,640
Vδ1+CD27+CD28+CD45RA-	MZ	-0,020	0,930	-0,449	0,416
Vδ1+CD27+CD28-	MZ	0,420	0,037	0,017	0,705
Vδ1+CD27-CD28+	MZ	0,261	0,208	-0,162	0,603
Vδ1+CD27-CD28-CD45RA-	MZ	0,301	0,143	-0,119	0,630
Vδ1+CD27-CD28-CD45RA+	MZ	0,377	0,063	-0,034	0,679
Vδ2+CD27+CD28+CD45RA+	MZ	0,832	< 0,0001	0,638	0,927
Vδ2+CD27+CD28+CD45RA-	MZ	0,549	0,006	0,174	0,785
Vδ2+CD27+CD28-	MZ	0,309	0,133	-0,110	0,635
Vδ2+CD27-CD28+	MZ	0,309	0,134	-0,111	0,635
Vδ2+CD27-CD28-CD45RA-	MZ	0,675	0,000	0,353	0,854
Vδ2+CD27-CD28-CD45RA+	MZ	0,305	0,157	-0,135	0,645
CD4+CD27+CD28+CD45RA+	DZ	0,186	0,474	-0,337	0,622
CD4+CD27+CD28+CD45RA-	DZ	0,054	0,837	-0,451	0,532
CD4+CD27+CD28-	DZ	0,342	0,179	-0,181	0,714
CD4+CD27-CD28+	DZ	0,552	0,022	0,081	0,821
CD4+CD27-CD28-CD45RA-	DZ	0,239	0,355	-0,287	0,655
CD4+CD27-CD28-CD45RA+	DZ	0,059	0,823	-0,447	0,536
CD8+CD27+CD28+CD45RA+	DZ	-0,025	0,926	-0,511	0,474
CD8+CD27+CD28+CD45RA-	DZ	0,427	0,088	-0,084	0,760
CD8+CD27+CD28-	DZ	0,363	0,152	-0,158	0,726
CD8+CD27-CD28+	DZ	0,367	0,148	-0,154	0,728
CD8+CD27-CD28-CD45RA-	DZ	0,422	0,092	-0,090	0,757
CD8+CD27-CD28-CD45RA+	DZ	-0,277	0,282	-0,677	0,250
Vδ1+CD27+CD28+CD45RA+	DZ	-0,055	0,873	-0,646	0,578
Vδ1+CD27+CD28+CD45RA-	DZ	0,582	0,066	-0,048	0,881
Vδ1+CD27+CD28-	DZ	-0,002	0,993	-0,494	0,491
Vδ1+CD27-CD28+	DZ	0,039	0,881	-0,462	0,522
Vδ1+CD27-CD28-CD45RA-	DZ	-0,313	0,221	-0,698	0,212
Vδ1+CD27-CD28-CD45RA+	DZ	0,069	0,794	-0,439	0,543
Vδ2+CD27+CD28+CD45RA+	DZ	0,103	0,694	-0,410	0,567
Vδ2+CD27+CD28+CD45RA-	DZ	0,434	0,082	-0,075	0,763
Vδ2+CD27+CD28-	DZ	0,178	0,495	-0,345	0,616
Vδ2+CD27-CD28+	DZ	0,385	0,127	-0,133	0,738
Vδ2+CD27-CD28-CD45RA-	DZ	-0,045	0,903	-0,641	0,584
Vδ2+CD27-CD28-CD45RA+	DZ	0,329	0,297	-0,320	0,768

A

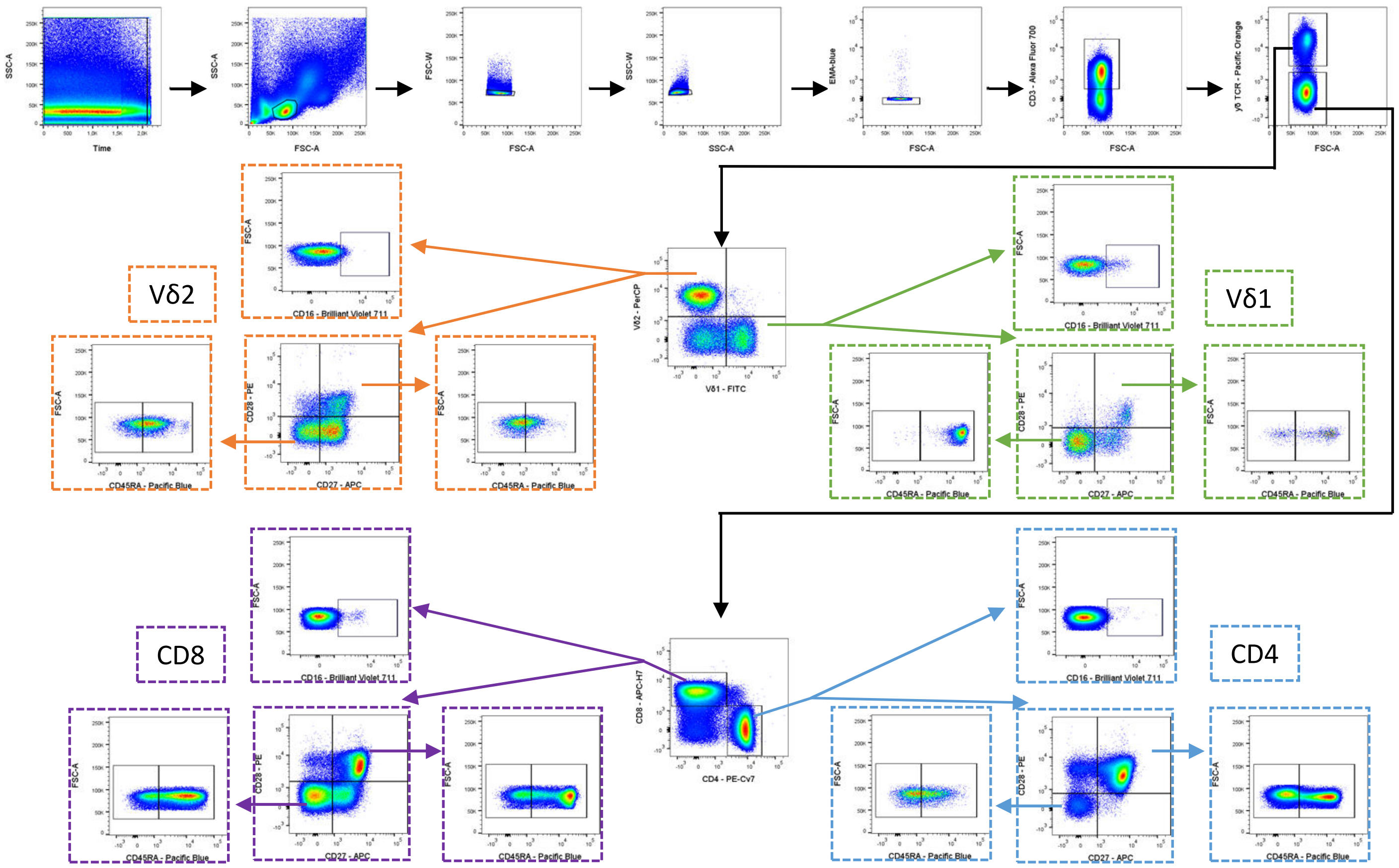

B

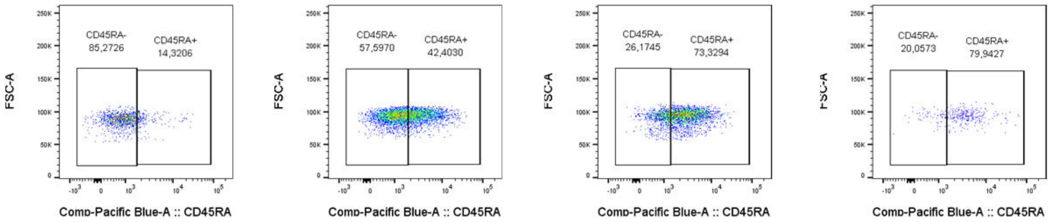

Supplementary Figure 1: Gating strategy for the analysis of γδ and αβ T cell phenotypes. **(A)** Data from a representative subject illustrates the here applied gating strategy that is similar to the one we suggested in the OMIP-20 [20]. **(B)** Donor heterogeneity in the expression of CD45RA on CD27+CD28+ Vδ2+ γδ T cells.

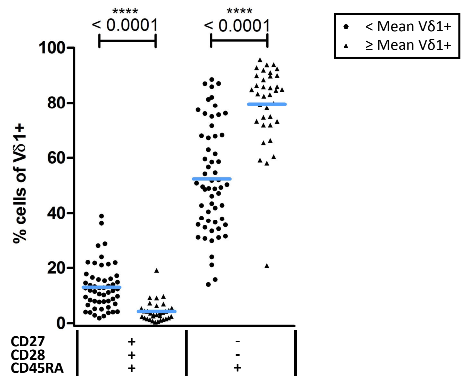

Supplementary Figure 2: Differentiation phenotypes of subjects with high/low frequencies of Vδ1. Distribution of early (CD27+CD8+CD45RA+) and late (CD27-CD28-CD45RA+) differentiated T cells of individuals with Vδ1 frequencies out of all CD3+ cells below/above the mean. Only early and very late differentiation phenotypes are plotted. Blue lines indicate the mean for each group. Statistical evaluation was performed using the Man-Whitney-U test with Bonferroni correction for multiple comparisons: p<0.0083 was defined as statistically significant.

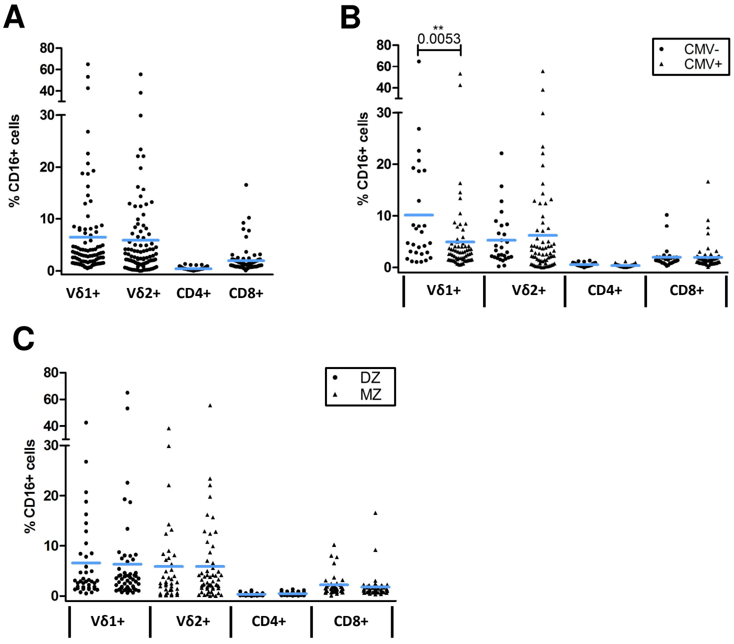

Supplementary Figure 3: CD16 expression on γδ and αβ T cell subsets. Frequencies of CD16 expressing Vδ1+, Vδ2+, CD4+ and CD8+ T cells **(A)**. Influence of CMV serostatus on CD16 expression **(B)**. Comparable distribution of CD16+ cells in MZ and DZ **(C).** Blue lines indicate the mean for each group. Statistical evaluation was performed using the Man-Whitney-U test with the Bonferroni correction for multiple comparisons: p<0.0125 was defined as statistically significant. Non significant results are not shown.

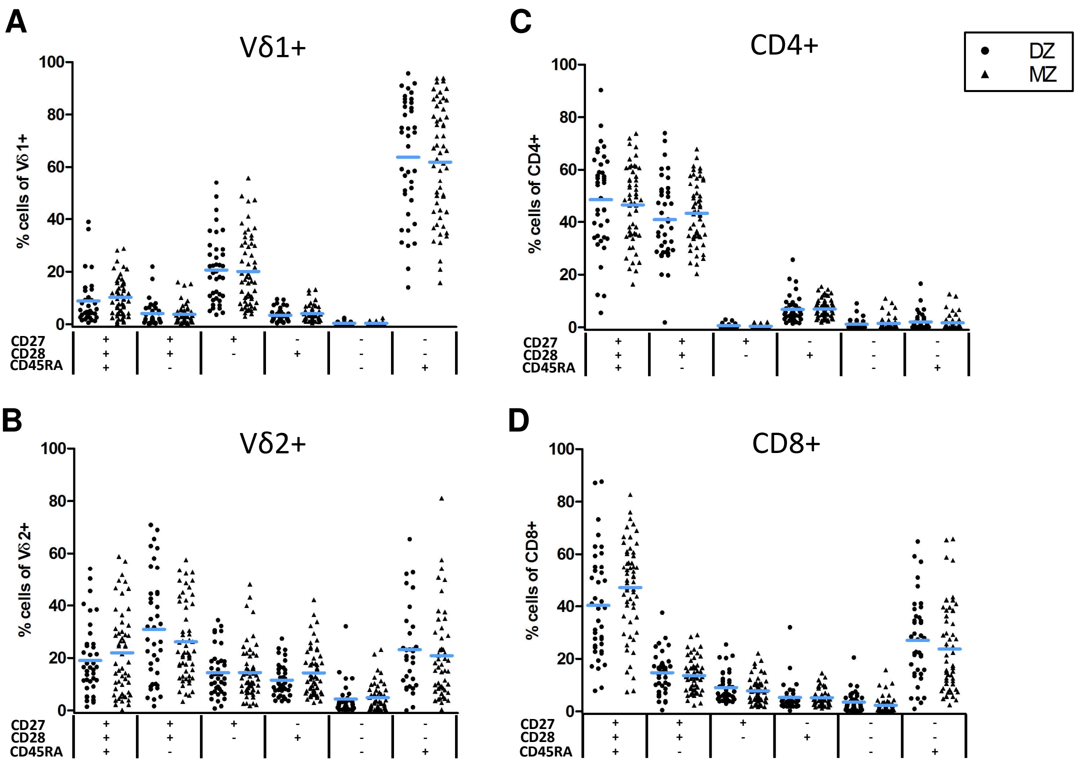

Supplementary Figure 4: Differentiation signatures of γδ and αβ T cell subsets are similar in monozygotic and dizygotic twins. The distribution of early, intermediate and late differentiated T cells was heterogeneous between donors and Vδ1+ **(A)**, Vδ2+ **(B)**, CD4+ **(C)** and CD8+ **(D)** T cells, but there were no significant differences between the groups of monozygotic (MZ) and dizygotic twins (DZ). Blue lines indicate the mean for each group (scatter plots). Statistical evaluation was performed using the Man-Whitney-U test with Bonferroni correction for multiple comparisons: p<0.0083 was defined as statistically significant. Non significant results are not shown.

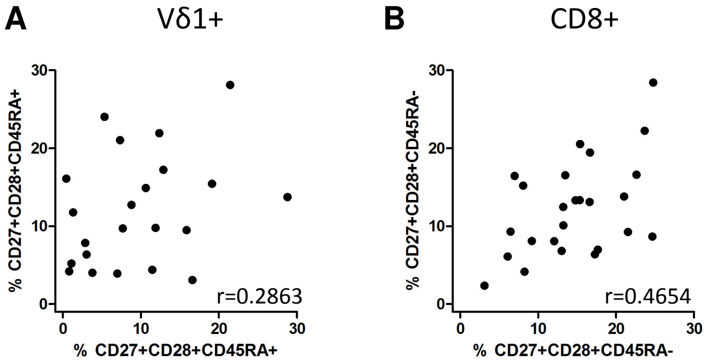

Supplementary Figure 5: Correlation of the T cell differentiation status in monozygotic co-twins. CD27+CD28+CD45RA+ Vδ1+ T cells **(A)** and CD27+CD28+CD45RA- CD8+ T cells **(B)** correlated only weakly in monozygotic (MZ) co-twins. The Spearman correlation coefficient r is shown.
